# Searching for protein variants with desired properties using deep generative models

**DOI:** 10.1186/s12859-023-05415-9

**Published:** 2023-07-21

**Authors:** Yan Li, Yinying Yao, Yu Xia, Mingjing Tang

**Affiliations:** 1 School of Information, Yunnan Normal University, Kunming, China; 2 National Key Laboratory of Crop Genetic Improvement and National Centre of Plant Gene Research, Huazhong Agricultural University, Wuhan, China; 3 Shenzhen Branch, Guangdong Laboratory of Lingnan Modern Agriculture, Genome Analysis Laboratory of the Ministry of Agriculture and Rural Affairs, Agricultural Genomics Institute at Shenzhen, Chinese Academy of Agricultural Sciences, Shenzhen, China; 4 Engineering Research Center of Sustainable Development and Utilization of Biomass Energy, Ministry of Education, Yunnan Normal University, Kunming, China; 5 School of Life Science, Yunnan Normal University, Kunming, China

**Keywords:** Protein engineering, Temporal convolutional network, Deep generative model, Variational autoencoder

## Abstract

**Background:**

Protein engineering aims to improve the functional properties of existing proteins to meet people’s needs. Current deep learning-based models have captured evolutionary, functional, and biochemical features contained in amino acid sequences. However, the existing generative models need to be improved when capturing the relationship between amino acid sites on longer sequences. At the same time, the distribution of protein sequences in the homologous family has a specific positional relationship in the latent space. We want to use this relationship to search for new variants directly from the vicinity of better-performing varieties.

**Results:**

To improve the representation learning ability of the model for longer sequences and the similarity between the generated sequences and the original sequences, we propose a temporal variational autoencoder (T-VAE) model. T-VAE consists of an encoder and a decoder. The encoder expands the receptive field of neurons in the network structure by dilated causal convolution, thereby improving the encoding representation ability of longer sequences. The decoder decodes the sampled data into variants closely resembling the original sequence.

**Conclusion:**

Compared to other models, the person correlation coefficient between the predicted values of protein fitness obtained by T-VAE and the truth values was higher, and the mean absolute deviation was lower. In addition, the T-VAE model has a better representation learning ability for longer sequences when comparing the encoding of protein sequences of different lengths. These results show that our model has more advantages in representation learning for longer sequences. To verify the model’s generative effect, we also calculate the sequence identity between the generated data and the input data. The sequence identity obtained by T-VAE improved by 12.9% compared to the baseline model.

## Introduction

As an essential life-sustaining biological macromolecule, proteins’ primary structure is composed of 20 different amino acids of variable lengths. Most existing stable proteins evolved from natural selection and continuous environmental changes over millions of years. Biological functions and structural uniqueness closely correlate to protein sequences. Since the 1970s, developments in protein engineering have aided researchers in exploring new protein variants in food [1], drug design [2]and industrial enzymes [3], and other fields to improve people’s lives. Protein sequences contain a wealth of information about evolution, function, fitness landscape, and so on. Generating proteins with better functional properties remains one of biology’s most important research directions. In traditional protein engineering, it is time-consuming to screen out better-performing variants needed by the industry from numerous random mutations of a single amino-acid sequence or recombination of natural homologous proteins.

Rapid developments in computer technology have made the use of machine learning in protein engineering an increasingly important research field [4–7]. Transfer learning uses many unlabeled protein sequences for pre-training to extract general proteins’ features and representations. The model is then fine-tuned with a small amount of labeled data, enabling the model to adapt to problem-specific downstream tasks [8–11]. Boomsma et al. [12] argue that extracting meaningful representations of raw protein sequence data into abstract, high-level, and low-dimensional spaces is critical to continued data exploration. Models such as ProGen [13], low-n [14], ESM-1v [15], and ECNet [16] have demonstrated that a joint optimization approach of “pre-training + fine-tuning” is feasible to obtain new variants of desired characteristics. However, it is challenging to train a well-performing language model, because large-scale protein language models often require massive amounts of data for training and are often limited by computing resources.

Unlike the embedding of models such as long short-term memory (LSTM), Transformer, and Resnet, the variational autoencoder (VAE) can clearly see phylogenetic separation in 2-dimensional latent space [12].Vincenzo et al. think that VAE models are more suitable for protein sequence covariation modelling and have advantages in modelling higher-order interactions [17]. In addition, Ding et al. [18] showed that latent space variables of VAE can capture the evolutionary relationship of homologous family sequences and simulate higher-order epistasis without exponentially increasing the number of parameters. This is conducive to exploring the protein fitness landscape and generating the necessary new sequences.

Generating new protein sequences is one of the VAE models’ most important functions. The model captures the evolutionary constraints of the training data by learning the representation of amino acid sequences and then searching the protein sequence space to find new sequences conforming to the evolutionary constraints. The primary goal of generative models is to generate variants that closely resemble the target protein. In this paper, we use sequence identity to measure the similarity of protein sequences. Sequence identity is the percentage of identical residues at corresponding positions in the same alignment length of two amino acid sequences. It can reflect the model’s ability to capture the type change of important sites in the sequence. For longer amino acid sequences, there are significant differences in sequence identity between generated and native. These differences are seen when the positions of many amino acid types on the generated sequences are changed, significantly reducing the similarity between the sequences. Therefore, we propose a model T-VAE based on a temporal convolutional network (TCN) [19].T-VAE consists of an encoder and a decoder. We use the TCN network structure in the encoder to expand the range of receptive neuron fields to capture the relationship between long-sequence sites. By comparing models and sequences of different lengths, T-VAE can better learn the representation of longer protein sequences. A continuous latent space allows the interpolation to follow the shortest Euclidean path between latent representations of the sequence. By changing the internal latent representation and decoding it, we can obtain new variants with higher fitness values in the protein sequence space. In addition, experiments show that the sequences generated by T-VAE have a higher sequence identity than the input sequences.

## Related work

### Sequence modeling

In recent years, research in computer vision and natural language processing (NLP) has focused on learning useful or unknown information from unlabeled data. In NLP tasks such as machine translation [20], speech recognition [21], and sentiment analysis [22], the encoded representation of input data has had a critical impact on the applicability and quality of the results of machine learning methods. Representations should preserve information relevant to the problem while reducing redundant data. Inspired by NLP, unlabeled protein sequences contain information about structure and function [23].Using the deep-learning network structure to learn the sequence-function mapping relationship effectively from protein sequences is necessary to improve the model’s ability to represent sequences. The sequence-to-sequence encoder model allows representation learning from raw data, so the potential representations of protein sequences can be learned in an unsupervised manner [24, 25]. As a neural network processing sequence data, a recurrent neural network (RNN) has more advantages in processing time-related sequence data than the traditional feedforward neural network [26].However, RNN is also prone to problems, such as gradient disappearance and gradient explosion. Furthermore, RNNs lose long-distance information that is dependent on longer sequences.

To process raw audio with long-range dependencies, Oord et al. [27] proposed an architecture of dilated causal convolutions, which exhibits a large receptive field and generates novel and highly realistic musical fragments. Bai et al. [19] proposed a structure for sequence modeling, the temporal convolutional network, which can take sequences of arbitrary length and map out fixed-length outputs. Temporal convolutional networks perform better than recurrent networks on different tasks and datasets while showing longer effective memory [19]. developed an autoregressive model that leverages causally dilated convolutional deep-generative networks to drive biological sequences, which captures functional constraints well and does not rely on explicit alignment structures. Kim et al. [28] used deep temporal convolutional networks to better predict mutational effects by capturing information from multiple sequence alignments with low, effective sequence numbers.

Convolutional neural networks have a parameter-sharing architecture, so they can learn to summarize the higher-level features across different sequence positions. To improve the sequences’ encoding ability, we utilize a layer of TCN modules in the encoding network structure. By enlarging the receptive fields of neurons in the network to capture relationships between distant sites, structural and co-evolutionary information in native protein sequences can be learned.

### Generative models in protein engineering

Machine learning is an efficient method for the selection of protein-directed evolution [4]. Neural networks learn protein sequences-function mappings from deep-mutation scanned data to predict previously undiscovered sequence functions [7]. Most existing generative models find the probability distribution of the data, and their training and sampling are excellent tests of their ability to represent the data and its probability distribution of high-dimensional features [29]. Deep generative models can be used to learn meaningful representations of protein sequences, assigning higher probabilities to protein sequences that satisfy desired criteria [5]. Goodfellow et al. [30] proposed a generative adversarial network (GAN) with generative and discriminative models. Models such as its variants DCGAN [31], CycleGAN [32], and StyleGAN [33] have achieved significant improvements in architecture and style transfer. To expand the sequence space of functional proteins, Repeatka et al. [34] designed a variant of the self-attention-based generative adversarial network ProteinGAN. ProteinGAN learns the evolutionary relationships and domain diversity between natural sequences from the complex multidimensional amino acid sequence space and generates new sequence variants with natural physical properties and domain diversity. Although the GAN-based model has achieved good results, model collapse and convergence difficulty may occur during the generative process. Compared with GAN, VAE can generate more stable features. After embedding and visualizing natural protein sequences in latent space, the distribution relationship of homologous sequences along the evolutionary direction can be observed [18]. VAEs impose lower bounds on the input probabilities, allowing a probabilistic interpretation of the results. The latent space encodes phylogenetic data and other possible features about proteins to guide the exploration of the protein sequence space [35]. Greener et al. [36] used the VAE model to generate desired properties to add potential copper and calcium binding sites to non-metal binding proteins. Hawkins-Hooker et al. [37] developed independent VAE models for the original and aligned sequences. They showed that versions trained by multiple sequence alignments improved the reproduction of functional constraints’ structures and statistical features, which are acquired and maintained when family members evolve.

Although most of the current work aims to generate “effective” sequences, we hope to use a generative model to optimize and eventually get “improved” sequences. In other words, the new variants we generated were not only “effective” proteins that functioned normally but also had more desirable fitness values. For example, when studying the stability of chimeric cytochrome proteins, we hope that the obtained protein variants have higher temperature resistance based on stability. It is currently difficult for feature representations to capture the relationship between distant sites for some proteins with longer sequences. As a result, the sequence identity between the generated and raw sequences was adequate. We propose the T-VAE model mainly to study how to search for new variants with higher fitness values in the sequence space of longer proteins. Theoretically, the model can generate new protein sequences from the known functional protein sequence space and minimize the need to test many nonfunctional protein sequence variants. Therefore, our model will reduce the difference between generated sequences and natural sequences for a small dataset, guiding the model to search for sequences with “improved” functional properties.

## Methods

**Fig. 1:**
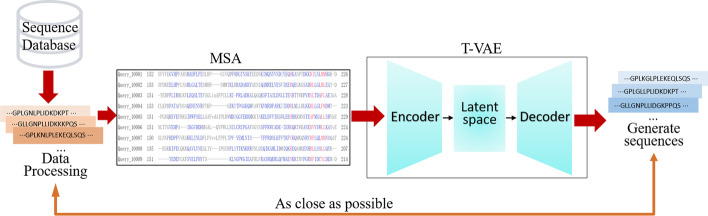
The workflow using a deep generative model. First, data is obtained from the database. After preliminary processing, the sequences are aligned by multiple sequence alignments. Next, they are fed into the deep generative model for training, generating the new sequences we need by sampling. The generated sequences should be as similar as possible to the training data

To improve the search for the protein we need in the protein sequence space, we propose the T-VAE model. Taking homologous family sequences with evolutionary relationships as research objects, we first processed or deleted the sequences we downloaded that did not meet the research requirements. Then, after multiple sequence alignments, the model trained the encoder and decoder simultaneously to obtain the parameters of the data distribution. Our encoding scheme attempted to capture prior domain knowledge about amino acids as the similarity between vectors. The representation output by the encoder was smoothed a priori to ensure that the internal latent representation could be changed or sampled from the prior distribution of latent vectors. Then, the resulting vector was fed to the decoder to obtain new protein sequences (Fig. 1). We used Gaussian process regression to predict new protein function values to verify whether the generated sequences had similar or better properties than the training data.

### T-VAE model details

**Fig. 2:**
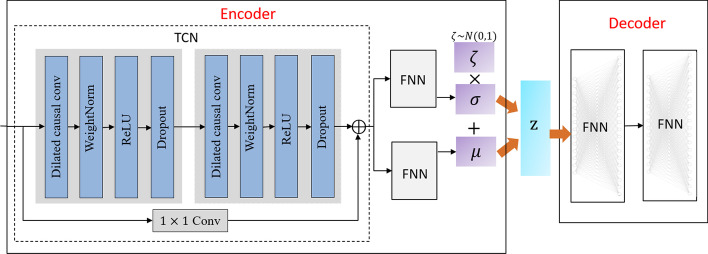
Network structure of T-VAE

The T-VAE model consists of an encoder and a decoder. As shown in Fig. 2, the encoder consists of a TCN module [19] and a layer containing a fully connected neural network. TCN uses a 1D fully convolutional network and causal convolutions for sequence encoding. The length of the amino acid sequence $S=(s_{1},s_{2},s_{3},...,s_{L})$ is *L*, and $s_{i}$ represents the amino acid type at the *i*th position in the sequence. Causal convolutions are input in the sequence according to the sequence of the proteins. The parameter information at $s_{i}$ is composed of the information of the *i*th site in the current layer and the information before the *i*th site in the previous layer, which means there will be no “missing connections” in the occurrence of historical information or future data. Given an input sequence $s_{1},...,s_{L}$ and the objective to output the corresponding sequence $\tilde{s_{1} },\dots , \tilde{s_{L} }$,a sequence modeling network is any function *f*:

1 $$\begin{aligned} \begin{aligned} \tilde{s_{1} },\dots , \tilde{s_{L} }=f(s_{1},...,s_{L}) \end{aligned} \end{aligned}$$

In a general convolutional network, the number of convolutional layers must be increased if the input variables must be considered immediately. However, this will cause problems such as gradient disappearance, complex training, and poor fitting. In the temporal convolutional network, the problem of long-term dependence is solved by introducing dilated convolution. Usually, deepening the network depth will expand the receptive field and improve the model’s performance so more information can be captured. Unlike traditional convolutional networks, dilated convolution injects holes into the convolution kernel, so the size of the effective window increases exponentially with the number of layers. The sampling rate is controlled by the expansion rate *d*, so the convolutional network exponentially expands the model’s receptive field with fewer layers. Therefore, when the dilated convolution ensures that the output size is constant, the range of information obtained is larger. The operation of the dilation convolution *F* on sequence *S* is defined as:

2 $$\begin{aligned} \begin{aligned} F\left( s \right) = \left( S*_{{d}}^{}f \right) \left( s \right) ={\textstyle \sum _{i=1}^{k}f\left( i\right) }\cdot S_{s-d_{i} } \end{aligned} \end{aligned}$$

where *d* is the dilation factor, *k* is the filter size, and $\left( {s-d_{i}} \right)$ accounts for the direction of the past. If the size of the convolution kernel used in the current layer is denoted as $k_{i}$ and the receptive field is denoted as $RF_{i}$,then the receptive field is calculated as follows:

3 $$\begin{aligned} \begin{aligned} RF_{i} = RF_{i-1}+\left( k_{i}-1 \right) \times S_{i} \end{aligned} \end{aligned}$$

where $RF_{i-1}$ denotes the size of the receptive field of the previous layer, and $S_{i}$ denotes the product of all layers except this layer. That is:

4 $$\begin{aligned} \begin{aligned} S_{i}= {\textstyle \prod _{j=1}^{i-1}}s_{j} \end{aligned} \end{aligned}$$

TCN also uses the residual connection to eliminate the problems of gradient disappearance and explosion that may exist in deep networks. The residual block turns the connection between layers into a residual structure, using dilated causal convolution, weight norm, dropout, and two layers of activation functions [19]. Weight norm and dropout are added to each layer to regularize the network. The ReLU activation functions are added to the residual blocks after the two convolutional layers. Except for the first layer’s input and the last layer’s output, the remaining layers in the residual block require the same input and output lengths. Considering that the network’s input and output channels may differ, a $1\times 1$ convolutional layer is also introduced into the residual block structure. The residual connection is the sum of the input *s* and the nonlinearly varying output ${\mathcal {F}}$, so the output $T_{O}$ in the residual block is:

5 $$\begin{aligned} \begin{aligned} T_{o}&=Activation\left( s+{\mathcal {F}}(s)\right) \end{aligned} \end{aligned}$$

where $Activation\left( \cdot \right)$ represents the activation function. The TCN network parameters in the model are: input dimension$=21\times L$, output dimension=100, kernel size=2, stride=1, padding=1, dilation=2, dropout=0.2. The fully connected layer network in the model uses the tanh function to activate neurons.

The encoder network can map a high-dimensional input *s* to a low-dimensional latent variable *z*. Given the observation sample *s*, the distribution of *z* can be deduced, that is $p\left( z\mid s \right)$. In high-dimensional continuous scenarios,$p\left( s \right)$ is intractable, because it requires marginalization over all possible values of *z*.

6 $$\begin{aligned} \begin{aligned} p\left( s\right) =\int p\left( z \right) p\left( s\mid z \right) dz \end{aligned} \end{aligned}$$

Via Bayes’ formula:

7 $$\begin{aligned} \begin{aligned} p\left( z\mid s \right) =\frac{p\left( s\mid z\right) p\left( z\right) }{p\left( s\right) } \end{aligned} \end{aligned}$$

This implies the intractability of the posterior $p\left( z\mid s \right)$. Thus, the distribution of $p\left( z\mid s \right)$ is approximated by a member $q\left( z\mid s \right)$ [38] of a parametric family of probability distributions, which is parameterized by the encoder neural network. We use Kullback–Leibler (*KL*) divergence to measure the distance between two distributions, denoted as $KL\left( q\left( z\mid s \right) \parallel p\left( z\mid s \right) \right)$, to find the optimal member $q\left( z\mid s \right)$ that is the best approximation of the true but unknown posterior.

Transform the above formula into:

8 $$\begin{aligned} \begin{aligned} logp(s)&=E_{q}\left[ logp(s,z) \right] -E_{q}\left[ logq(z\mid s)\right] +KL(q(z\mid s)\parallel p(z\mid s)) \end{aligned} \end{aligned}$$

The optimization objective of variational autoencoders is the evidence lower bound objective (ELBO):

9 $$\begin{aligned} \begin{aligned} ELBO&=E_{q}\left[ logp(s,z) \right] -E_{q}\left[ logq(z\mid s)\right] \end{aligned} \end{aligned}$$

It is easy to prove the variational lower bound:

10 $$\begin{aligned} \begin{aligned} ELBO&=E_{q}(log(p(s\mid z)))-KL(q(z\mid s)\parallel p(z)) \end{aligned} \end{aligned}$$

where the first term represents the reconstruction ability of the model from the latent space, and the second term is the KL divergence between the approximate distribution $q\left( z\mid s \right)$ and the prior distribution *p*(*z*) . Then, from formula $\left( 8 \right)$ and formula $\left( 9 \right)$, it is obtained that

11 $$\begin{aligned} \begin{aligned} logp(s)&=ELBO+KL(q(z\mid s)\parallel p(z\mid s)) \end{aligned} \end{aligned}$$

And because $KL(q(z\mid s)\parallel p(z\mid s))\ge 0$, then:

12 $$\begin{aligned} \begin{aligned} logp(s)\ge ELBO=E_{q}(log(p(s\mid z)))-KL(q(z\mid s)\parallel p(z)) \end{aligned} \end{aligned}$$

Finally, we need to maximize $logp\left( s \right)$,equivalent to maximizing the evidence lower bound. Therefore, $p\left( s \right)$ can be approximated using ELBO.

The encoder network learns the mean and variance of the latent variable probabilities to obtain the distribution parameters of the data. We randomly sample on the standard normal distribution to get the latent variable *z*. Each sampled point corresponds to a Gaussian distribution $N\left( \mu ,\sigma \right)$. The decoder reconstructs an approximate probability distribution of the original data based on the probability distribution of hidden variables generated by the encoding network. The decoder is responsible for restoring the data with the least loss, remapping the low-dimensional hidden variables into high-dimensional output. The algorithm flow of the T-VAE model is as in Algorithm 1.
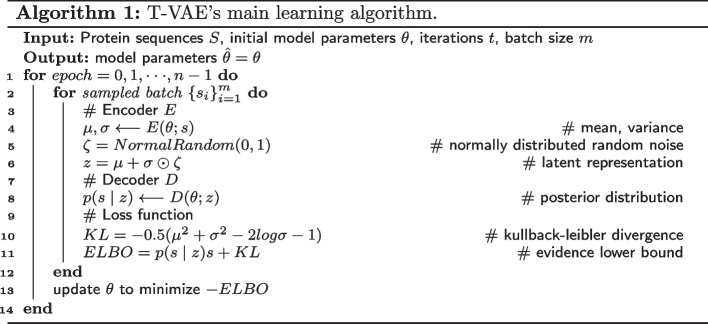


### Gaussian process regression to predict protein fitness values

New sequences are generated when our model is pre-trained with homologous family sequences. We use the sequence data (unlabeled) of the target protein family to pre-train the model so that the T-VAE model can learn the characteristics of the family’s co-evolutionary relationship. Then, fine-tune the T-VAE model using partial target protein data (labeled, including protein sequence and fitness value). These labels are not used when training the T-VAE but are used in the Gaussian process regression prediction model to evaluate the quality of the generated sequence.

Given a dataset $Q =\left\{ (s^{i},y^{i})\mid i=1,2,\cdots ,n\right\}$ consisting of *n* $M-dimensional$ input data *s* and corresponding labels y.Gaussian process regression assumes a prior distribution to infer the implicit function $g: R^{M}\longrightarrow R$ so the fitness value corresponding to the new sequences can be predicted when we generate the new sequences. The implicit function can be uniquely determined by the mean function and the covariance function:

13 $$\begin{aligned} \begin{aligned} g(s)&=GP(\mu ,k(s,{s}')) \end{aligned} \end{aligned}$$

Where $\mu$ is the mean and $k(\cdot ,\cdot )$ is the kernel function. Let the latent space *z* be the feature vector of the sequence. Use a radial basis function kernel:

14 $$\begin{aligned} \begin{aligned} k(z^{1},z^{2})&=\sigma _{g}^{2}exp\left( -\frac{1}{2}\frac{\left\| z^{1}-z^{2} \right\| ^{2}}{\lambda ^{2}} \right) \end{aligned} \end{aligned}$$

Maximize the likelihood of the Gaussian process model to estimate the label data to find the variance parameter $\sigma ^{2}$ and the length scale parameter $\lambda$.

## Results

We designed a set of comparative experiments to verify that the T-VAE model could learn protein sequence encodings effectively. We downloaded public data from the Pfam database [39]to verify the T-VAE model’s encoding effect. We collected and processed the downloaded dataset and then used the processed data to train the T-VAE model. When the model reached convergence, we embedded all protein sequences into the latent space and visualized the distribution characteristics. We used Pearson’s correlation coefficient and mean absolute deviation (MAD) as evaluation indicators. Then, we used T-VAE’s generative network to generate numerous protein sequences from the latent space and the Gaussian process regression model to predict the fitness value corresponding to the generated sequence and screened out new variants with higher fitness values.

### Data

The homologous protein family has statistical characteristics that reflect the shared evolutionary history and related structures and functions of family members [37]. Cytochrome P450 (P450) is the most widely used catalyst in plants, which can be used to synthesize many specialized metabolites with diverse structures. It is also a key enzyme in the drug metabolic process, providing a valuable resource in the development of new drugs [40]. Therefore, we chose the sequences of the cytochrome P450 protein family (PF00067) as our research object and downloaded the sequence of the entire family from the Pfam database [39]. After downloading the data, we needed to process or delete the data that did not meet the training requirements. The process was as follows.

- Delete the gap sites of “.” and “-” in each sequence.
- Delete sequences in the family where the gap exceeded 20% of the total length of the sequences.
- Remove repetitive sequences.
- Sequence alignment.

After processing, the data that could be used for training had a total of 57,356 sequences, and the sequence length consisted of 426 amino acids. The types of amino acids were represented by numbers from 1 to 20, and the gaps in MSA were represented by 0. We weighted MSA sequences in protein families using a position-based sequence weighting method [18] to reduce the distributional bias in which some species are more easily detected than others. The input was represented as a $21\times L$ binary matrix.

### Models training

We used the Pytorch framework to build the model architecture. To support matrix operations for deep learning, the GPU we use is NVIDIA Tesla T4 16 G. The server environment is Windows 10 operating system of 64-bit, which has a CPU of Intel(R) Xeon(R) Gold 5117 CPU @ 2.00GHz 2.00 GHz.Under the above hardware and software conditions, we trained and tested the representation learning ability and sequence generation ability of the proposed TVAE model. As a control, full connected-VAE (F-VAE) and LSTM-VAE (L-VAE) models are also tested.

### Experimental results and analysis

#### Latent space representation of protein sequences

**Fig. 3:**
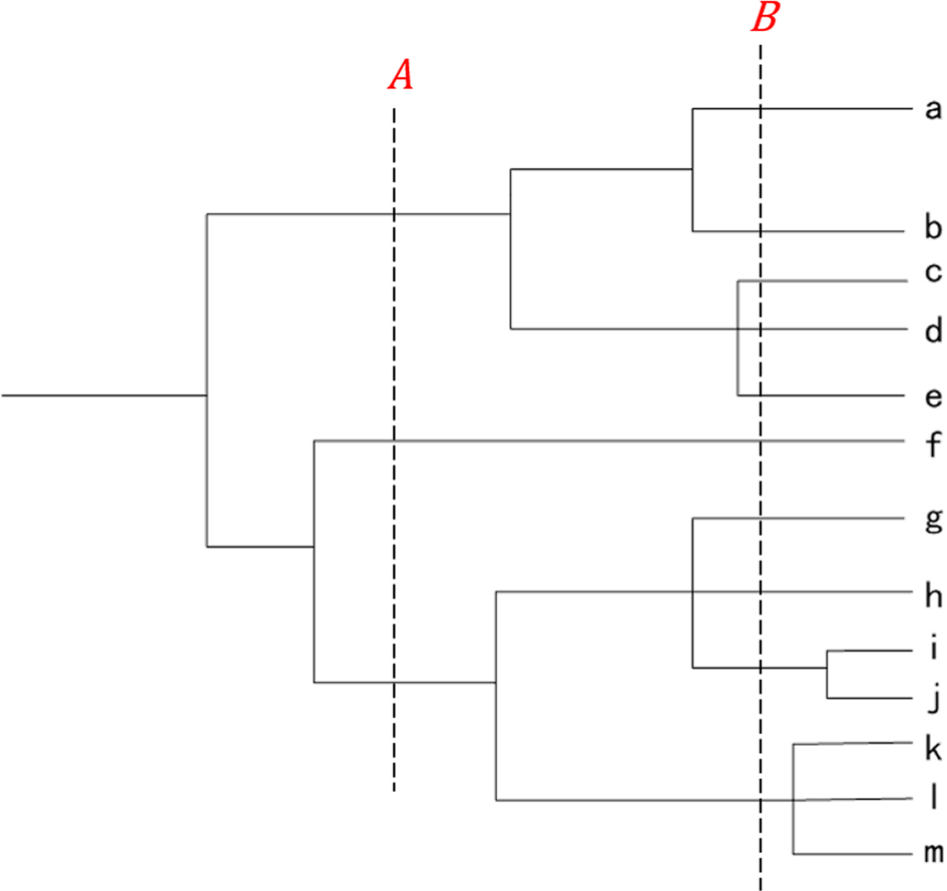
A schematic representation of the phylogenetic tree

**Fig. 4:**
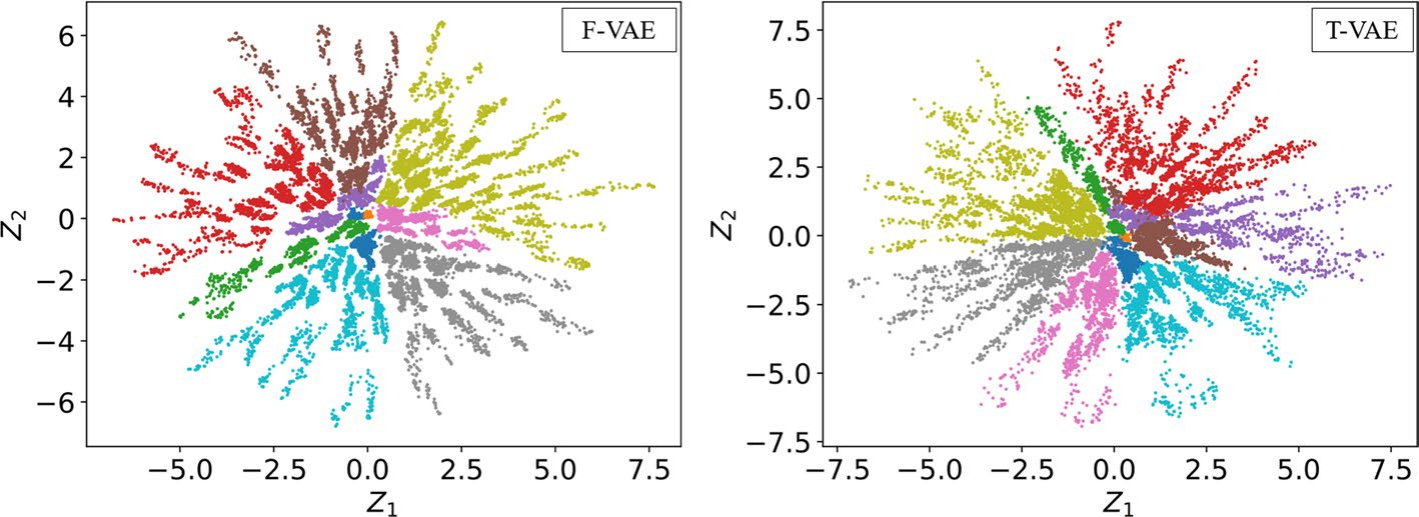
Representations of protein sequences in latent space

Proteins within a family have statistical characteristics that reflect evolutionary patterns among members [37]. In general, VAE’s simple architecture can learn the evolution of sequences in the family. Figure 3 is a schematic representation of the phylogenetic tree, where *A* and *B* represent two evolutionary time points from the root node. The value size at the time node generally represents the evolutionary distance from the root node. The protein sequences of the simulated phylogenetic tree with 10,000 leaf nodes [18] were all embedded in the F-VAE and T-VAE (Fig. 4). For visualization, we used a two-dimensional latent space. Figure 4 shows that the distribution of data embedded in the two models is similar; both are star-shaped structures with spikes. When the evolutionary distance is set to 2.4, the sequences are grouped, and the sequences in the same group have the same color. We can observe that the distribution of the two scatter plots is similar, and the points of the same color are clustered in the same area. It shows that the same sequences with evolutionary relationships are distributed in the same region. We can interpolate in its latent space to obtain similar variant sequences related to a specific protein. The new sequences are likely to be evolutionarily related to the original sequence.

**Fig. 5:**
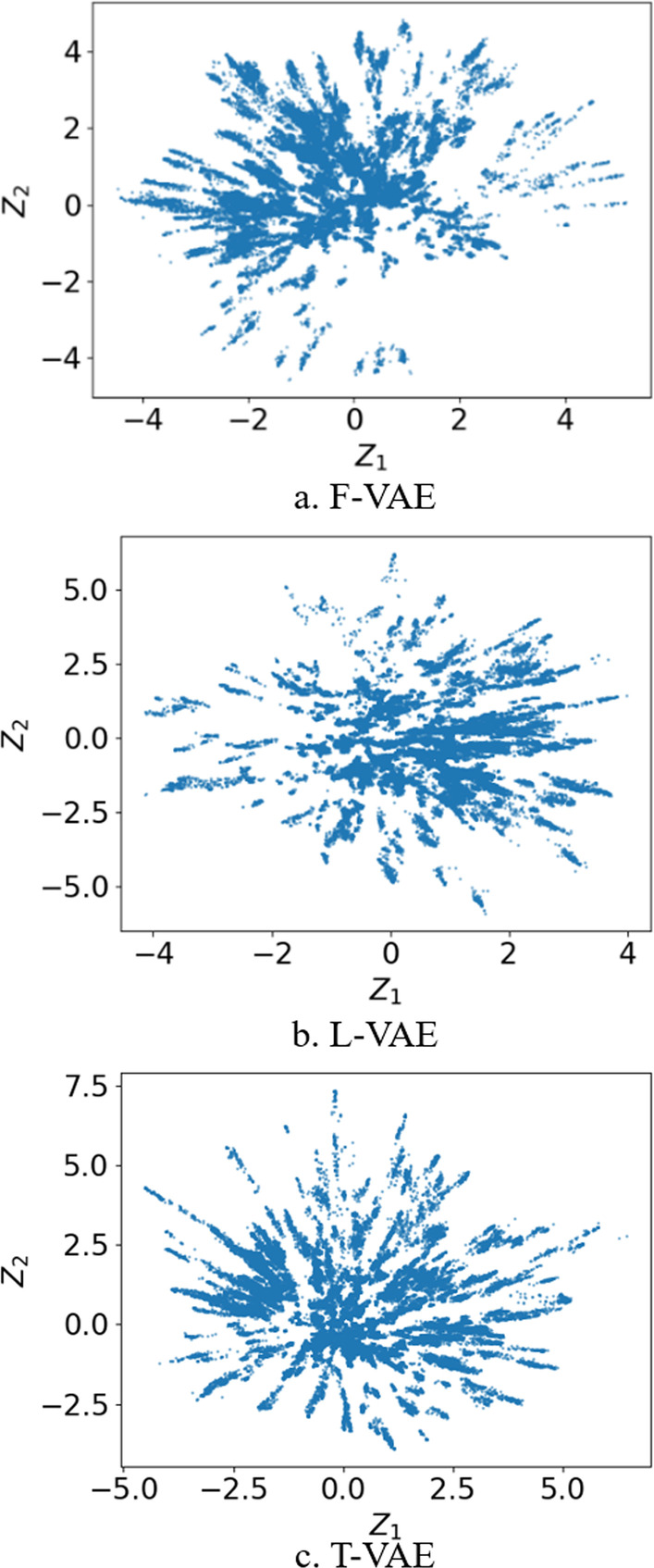
The distribution of cytochrome P450 family sequences embedded in the two-dimensional latent space of **a** F-VAE, **b** L-VAE, and **c** T-VAE

We used the phylogenetic reconstruction method to embed the phylogenetically related CYP450 family sequences into a two-dimensional latent space (Fig. 5) to observe the distribution of sequences embedded in the latent space. After visualization, we observed that the data are almost centered on the coordinate (0,0) and spread out in peak-like shapes. To investigate the effect of T-VAE on the distribution of encoded sequences in latent space, we set up two models for comparative experiments, F-VAE and L-VAE. The F-VAE encoder consists of a two-layer fully connected feedforward neural network [18], and the L-VAE encoder consists of an LSTM network module and a fully connected feedforward neural network layer. The decoders’ network structure in these two models is the same as that of the decoder in T-VAE. Embedding the P450 family data into these two models’ two-dimensional latent space (Fig. 5a, b), we can also see the spikes extending from the center to the periphery.

**Fig. 6:**
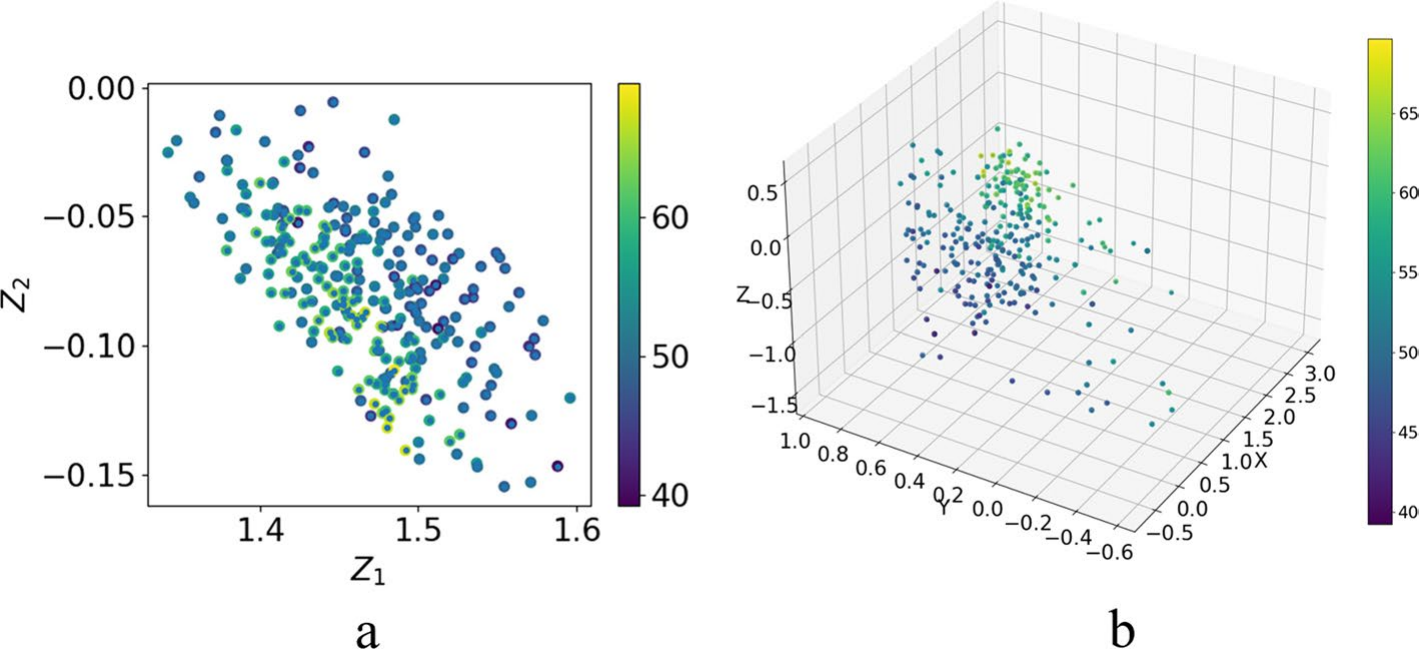
Visual distribution of 278 chimeric cytochrome P450 sequences embedded in **a** two-dimensional and **b** three-dimensional latent space

We used 278 chimeric cytochromes with fitness labels [18, 41] (Fig. 6) to view the distribution of data embedded in the two-dimensional and three-dimensional latent space of the T-VAE model microscopically. The colors of the sequences represent the value $T_{50}$ (the temperature at which 50% of the protein is irreversibly inactivated). In the two-dimensional latent space, the data with higher $T_{50}$ values are distributed on the lower-left side. In the two-dimensional visualization graph, the data with higher $T_{50}$ values are distributed on the lower-left side. In the three-dimensional latent space, it can be seen that the data distribution has specific rules. The data distribution is based on the high and low $T_{50}$values in the latent space. Sequences with low or high values are concentrated in specific regions. It shows that the protein sequence has a specific positional relationship distribution in the latent space, which can be observed. If interpolation is performed near a sequence with excellent fitness, the obtained sequence fitness is likely similar or higher. We hope to use this on-site visualization to search for more high-performing proteins directly in the vicinity of high-performing species. This provides us with a theoretical basis on which to search for new sequences with improved properties in the protein sequence space.

#### T-VAE has the advantage of encoding protein sequences.

**Fig. 7:**
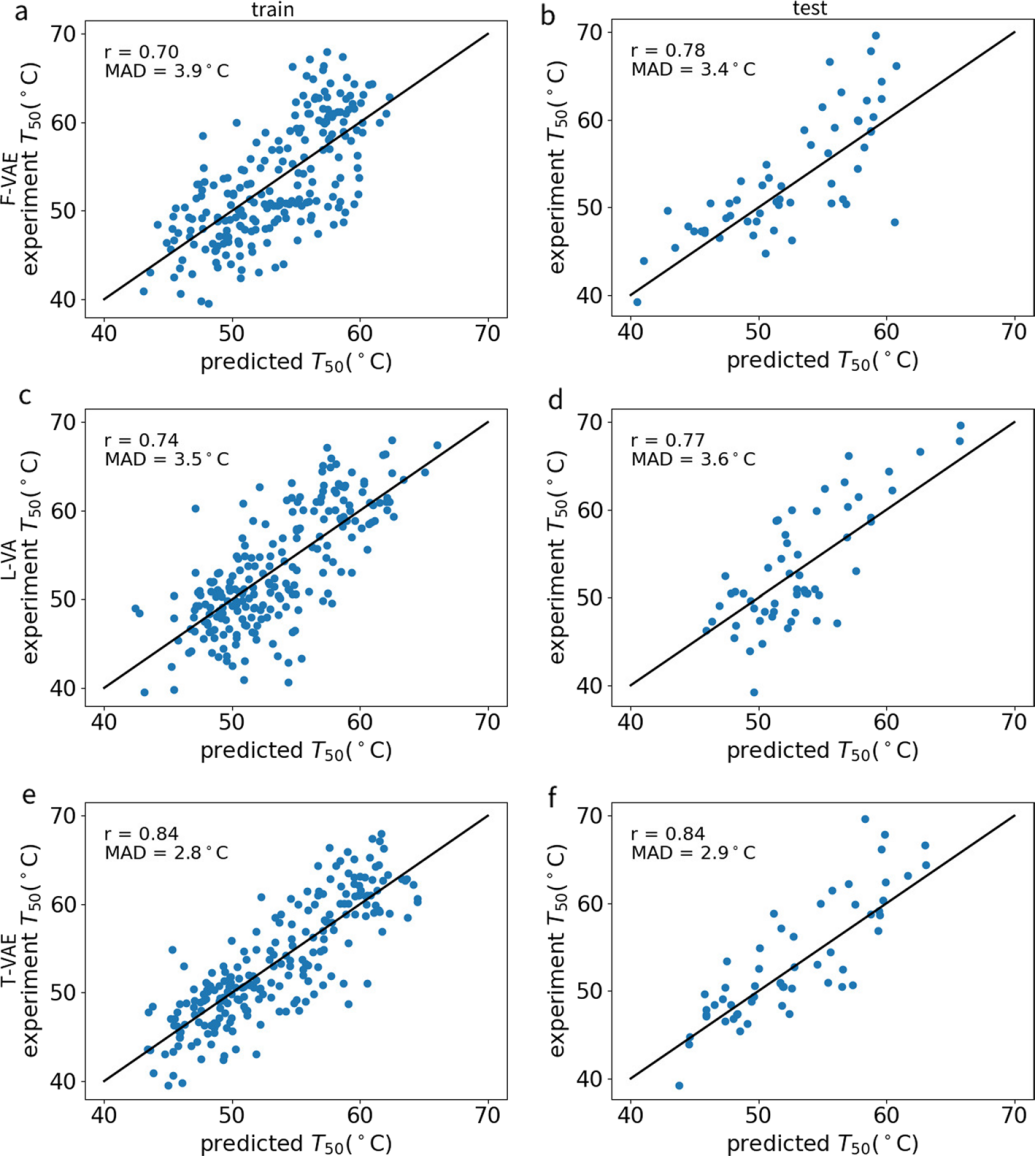
Performance of Gaussian process regression for predicting cytochrome $T_{50}$ values

Protein fitness here refers to protein properties contributing to the normal functioning of a protein, such as protein stability and fluorescence, among others. Generating proteins with desired properties is primarily about searching for valuable protein variants. We used Gaussian process regression to predict the possible fitness values of the generated sequences. The experiment measured 278 chimeric cytochrome P450 sequences [42] input into the F-VAE, L-VAE, and T-VAE models to obtain the sequence encoding representation. Then, they entered the Gaussian process regression model with the corresponding $T_{50}$ value to study the extent to which the model quantified sequence features (Fig. 7). There were 222 training data and 56 test data. After training, the F-VAE Pearson correlation coefficient was 0.70, the MAD was 3.9$\,^{\circ }$C (Fig. 7a),and the test set Pearson correlation coefficient was 0.78. The MAD was3.4$\,^{\circ }$C (Fig. 7b). The Pearson correlation coefficient obtained by L-VAE was 0.74, the MAD was 3.5$\,^{\circ }$C (Fig. 7c), and the Pearson correlation coefficient of the test set was 0.77. The MAD was 3.6$\,^{\circ }$C (Fig. 7d). The Pearson correlation coefficient obtained by T-VAE was 0.84, the MAD was 2.8$\,^{\circ }$C (Fig. 7e), and the Pearson correlation coefficient of the test set was 0.84. The MAD was 2.9$\,^{\circ }$C (Fig. 7f).The predicted data after T-VAE model training had a higher correlation with the experimental data, and the MAD value was lower. The T-VAE model improved the encoding representation of long sequences and thereby improved the accuracy of the Gaussian process regression prediction, which provided the premise for us to predict the fitness value of the generated sequences.

We also used the small proteins (pin1 WW-domain, hYAP65 WW-domain, villin HP35, BBL, and 116 mutants of these proteins) and green fluorescent protein data in TAPE [23]to predict the stability landscape and fluorescence landscape. We used Gaussian process regression to predict the performance of the stability landscape on the training and test sets of small protein sequences. We also used it to predict the performance of the fluorescence landscape on the training and test sets of the green fluorescent protein sequence. The small proteins each consisted of 50 residues. There were 53,614 training sets and 12,851 test sets. The fluorescent proteins consisted of 237 residues. There were 21,446 training sets and 5,362 test sets. We reported Pearson correlation coefficients and MAD values between truth values and predicted values for fitness landscapes. Table 1 shows that as the protein length increased, we achieved better results on both the training and test sets. The Pearson correlation between the predicted fitness value and the truth value improved significantly, and the MAD value significantly decreased. This shows that adding the TCN module in the encoder network can capture the relationship between long sequence sites, and the effect of encoding representation is improved.

**Fig. 8:**
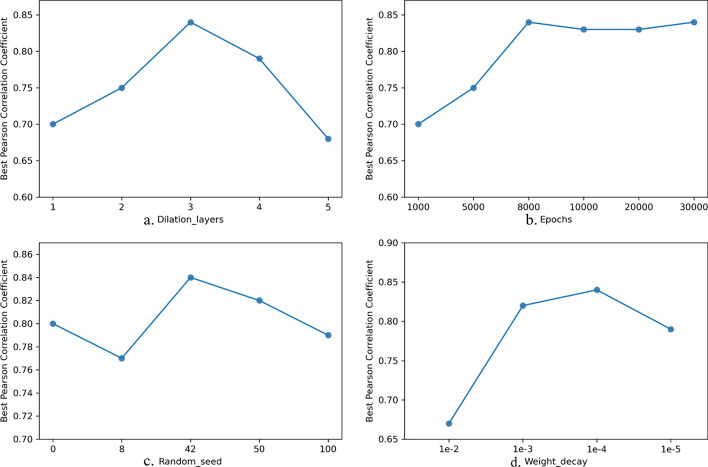
Pearson correlation coefficient between the Gaussian process prediction and the experimental $T_{50}$ data of the chimeric cytochrome P450 sequence test set when using different parameters. **a** Dilation layers; **b** Epochs; **c** Random seed; **d** Weight decay

We used the fixed variable method to try to adjust multiple hyperparameters, and the best results of each parameter on the test set are shown in Fig. 8. The number of dilation layers did not improve as it increased. In our experiment, the correlation between the Gaussian process regression prediction and the experimental $T_{50}$ data from the chimeric cytochrome P450 sequence test set was best when $layers = 3$ (Pearson’s $r = 0.84$ ). As the number of iterations increased, the model’s training effect improved. When epochs reached 8,000, the model’s training tended to be stable. Random seeds were selected in the group of $\{ 0,8,42,50,100 \}$. When the random seed took 42, the model training obtained the optimal value. To avoid model overfitting, we added weight decay to the neural network. The weight decay factor was selected from the set of values $\{ 0.01,0.001,0.0001,0.00001 \}$.The model trained best when weight $decay = 0.0001.$

**Table 1:** Pearson correlation coefficients and MAD values between ground truth and predicted fitness landscapes on proteins of different lengths

		Training set		Test set	
	Length	Pearson	MAD	Pearson	MAD
Small proteins	50	0.53	0.31	0.51	0.34
Fluorescent protein	237	0.79	0.27	0.75	0.32
Chimeric cytochrome	426	0.84	2.8	0.84	2.9

#### Generating new protein sequences.

**Table 2:** Parameter information for pre-training and fine-tuning of F-VAE and T-VAE

	Data_nums	Epochs	Weight_decay	Dim_z	Hidden_layer	Seed
Train_F-VAE	57356	20000	0.0001	3	100	42
Finetune_F-VAE	39	8000	0.0001	3	100	42
Train_T-VAE	57356	20000	0.0001	3	100	42
Finetune_T-VAE	39	8000	0.0001	3	100	42

We further evaluated the model-generated sequences’ performance using 39 labeled chimeric cytochrome fine-tuned F-VAE and T-VAE models.Table 2 shows the parameters required to train the two models. We sampled 10,000 points around the distribution with the highest fitness $T_{50}$ and decoded them into 10,000 protein sequences through a generator network. We used the Bio.pairwee2 module in Biopython to compute global and local identity alignments between the sequence with the highest $T_{50}$ value and all generated sequences. The average identity of the F-VAE sequence was 71.3% (three digits are reserved), and the maximum identity was 75.5%. The average identity obtained by T-VAE was 84.2%, and the maximum identity was 93.8% (sequence ID: Generate_92). The results of the identity calculation between sequences showed that the T-VAE model had more advantages in the feature extraction of amino acid sequences, and the difference between the generated sequence and the natural sequence was lower. We used Gaussian process regression to predict the $T_{50}$ values corresponding to 10,000 new sequences generated by the T-VAE model. We screened out sequences with $T_{50}$ values greater than the maximum corresponding to natural sequences (max=69.7$\,^{\circ }$C) and less than 72$\,^{\circ }$C. We screened 61 sequences, of which the maximum identity was 86.1% (sequence ID: Generate_5322), and the average identity was 84.3%.

**Fig. 9:**
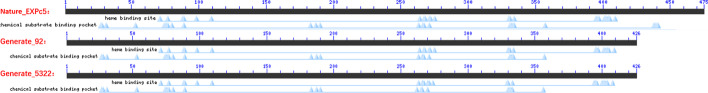
The natural sequence (Nature_EXPc5) and the generated sequences (Generate_92 and Generate_5322) all have heme binding sites, and the chemical substrate binding pockets and corresponding sites are nearly similar

The domain is not only the stable evolutionary unit of a protein but also an important part of structure and function prediction [43], which plays an important role in completing its physiological function [44]. We used the Conserved Domain Database (CDD) in NCBI to analyze the domains of the generated sequences. The conserved domains of the generated sequences of the identity of the top 100 were predicted using the Batch CD-search tool. We searched for matching results using the default database PSSMs. The specific hits of the generated sequences were found to belong to CYP120A1, the short name of a conserved domain cytochrome_P450 superfamily. We also predicted the conserved domains of generated sequences (Generate_92 and Generate_5322) and natural sequences (Nature_EXPc5) using the CD-search tool. The conserved domains of the three sequences were found to be highly identical, and all of them retained key substrate-binding and catalytic residues (Fig. 9), indicating that the generated sequences possessed the same functional domains as the natural sequences.

Subcellular localization is critical for predicting the likely function of the generated sequences [45]. To further verify that the T-VAE model can learn the location of natural sequences in the biological environment to help us make a preliminary judgment on the function of the generated protein, we used deep learning-based Deeploc$-$1.0 [46]to compare the generated sequences (Generate_92 and Generate_5322) with the natural sequence (Nature_EXPc5) for subcellular localization analysis.

**Fig. 10:**
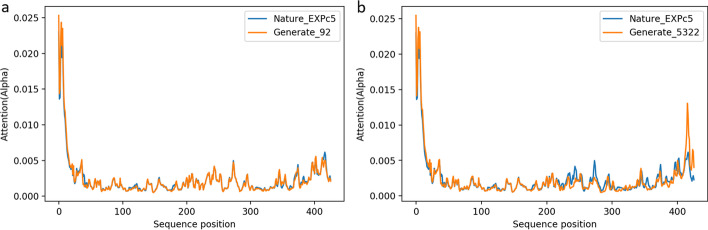
Nuclear signal localization comparison of generated and natural sequences. The orange line represents the important positioning of the generated sequence site, and the blue line represents the important positioning of the natural sequence site

Protein encoding uses BLOSUM62, and localization prediction shows that both new and natural sequences are mainly distributed in Cytoplasm and are soluble proteins (Table 3). In the comparison diagram of nuclear signal localization in Fig. 10, the nuclear localization signal is roughly at the initial position of the sequence, and the position of the nuclear localization signal of the generated sequences and the natural sequence almost coincides. It shows that the sequences generated by our model retain important functional sites.

**Table 3:** Subcellular localization analysis of natural sequence and generated sequences with the highest identity using Deeploc$-$1.0

	Cytoplasm (%)	Soluble(%)
Nature_EXPc5	0.7974	0.9594
Generate_92	0.8113	0.9655
Generate_5322	0.7577	0.9466

## Conclusion

We have shown that sequence encoding affects data distribution. By comparing different models, we have proven that adding a TCN module to the encoder network can improve the performance of sequence representation learning. We compared the coding performance of different length sequences on T-VAE. The experimental results show that our model has a more robust feature extraction ability for long sequences; that is, the model can extract more information on the site. In the generation task, the sequences generated by the T-VAE model have a higher similarity to natural sequences, indicating that we can search for better fitness near well-characterized protein representations based on the positional relationship of the data distribution in the latent space. This reduces the scope of the search in the huge protein sequence space and provides a new approach for deep learning in protein engineering and a feasible solution for generating directed evolution target protein variants quickly and efficiently.

## Data Availability

The cytochrome P450 data used in this paper are available in the Pfam database (http://pfam.xfam.org) with accession ID (PF00067). The experimental $T_{50}$ values for 278 P450 sequences are downloaded from the supplementary dataset of refs. [18]( https://www.nature.com/articles/s41467-019-13633-0).

## Abbreviations

TCN: Temporal convolutional network
VAE: Variational autoencoder
NLP: Natural language processing
RNN: Recurrent neural network
GAN: Generative adversarial networks
LSTM: Long short-term memory
GP: Gaussian process
ELBO: Evidence lower bound objective

## References

[1] Loveday SM (2019). Food proteins: technological, nutritional, and sustainability attributes of traditional and emerging proteins. Annu Rev Food Sci Technol.

[2] Kuhn B, Guba W, Hert J, Banner D, Bissantz C, Ceccarelli S, Haap M, Kuglstatter A, Lerner C (2016). A real-world perspective on molecular design: Miniperspective. J Med Chem.

[3] Cai T, Sun H, Qiao J, Zhu L, Zhang F, Zhang J, Tang Z, Wei X, Yang J, Yuan Q (2021). Cell-free chemoenzymatic starch synthesis from carbon dioxide. Science.

[4] Yang KK, Wu Z, Arnold FH (2019). Machine-learning-guided directed evolution for protein engineering. Nat Methods.

[5] Wu Z, Johnston KE, Arnold FH, Yang KK (2021). Protein sequence design with deep generative models. Curr Opin Chem Biol.

[6] Ding W, Nakai K, Gong H (2022). Protein design via deep learning. Brief Bioinform.

[7] Gelman S, Fahlberg SA, Heinzelman P, Romero PA, Gitter A (2021). Neural networks to learn protein sequence-function relationships from deep mutational scanning data. Proc Natl Acad Sci.

[8] Heinzinger M, Littmann M, Sillitoe I, Bordin N, Orengo C, Rost B. Contrastive learning on protein embeddings enlightens midnight zone at lightning speed. bioRxiv, 2021. 10.1093/nargab/lqac043 PMC9188115 35702380

[9] Rives A, Meier J, Sercu T, Goyal S, Lin Z, Liu J, Guo D, Ott M, Zitnick CL, Ma J (2021). Biological structure and function emerge from scaling unsupervised learning to 250 million protein sequences. Proc Natl Acad Sci.

[10] Agarwal V, Reddy N, Anand A. Unsupervised representation learning of dna sequences; 2019. arXiv preprint arXiv:1906.03087.

[11] Elnaggar A, Heinzinger M, Dallago C, Rihawi G, Wang Y, Jones L, Gibbs T, Feher T, Angerer C, Steinegger M, et al. Prottrans: towards cracking the language of life’s code through self-supervised deep learning and high performance computing; 2020. arXiv preprint arXiv:2007.06225. 10.1109/TPAMI.2021.3095381 34232869

[12] Detlefsen NS, Hauberg S, Boomsma W (2022). Learning meaningful representations of protein sequences. Nat Commun.

[13] Madani A, McCann B, Naik N, Keskar NS, Anand N, Eguchi RR, Huang P-S, Socher R. Progen: Language modeling for protein generation; 2020. arXiv preprint arXiv:2004.03497.

[14] Biswas S, Khimulya G, Alley EC, Esvelt KM, Church GM (2021). Low-n protein engineering with data-efficient deep learning. Nat Methods.

[15] Meier J, Rao R, Verkuil R, Liu J, Sercu T, Rives A (2021). Language models enable zero-shot prediction of the effects of mutations on protein function. Adv Neural Inf Process Syst.

[16] Luo Y, Jiang G, Yu T, Liu Y, Vo L, Ding H, Su Y, Qian WW, Zhao H, Peng J (2021). Ecnet is an evolutionary context-integrated deep learning framework for protein engineering. Nat Commun.

[17] McGee F, Hauri S, Novinger Q, Vucetic S, Levy RM, Carnevale V, Haldane A (2021). The generative capacity of probabilistic protein sequence models. Nat Commun.

[18] Ding X, Zou Z, Brooks CL (2019). Deciphering protein evolution and fitness landscapes with latent space models. Nat Commun.

[19] Bai S, Kolter JZ, Koltun V. An empirical evaluation of generic convolutional and recurrent networks for sequence modeling; 2018. arXiv preprint arXiv:1803.01271.

[20] Bahdanau D, Cho K, Bengio Y. Neural machine translation by jointly learning to align and translate; 2014. arXiv preprint arXiv:1409.0473.

[21] Karita S, Chen N, Hayashi T, Hori T, Inaguma H, Jiang Z, Someki M, Soplin NEY, Yamamoto R, Wang X, *et al.* A comparative study on transformer vs rnn in speech applications. In: 2019 IEEE automatic speech recognition and understanding workshop (ASRU), 2019:449–456. IEEE.

[22] Hu D, Wei L, Huai X. Dialoguecrn: Contextual reasoning networks for emotion recognition in conversations; 2021. arXiv preprint arXiv:2106.01978.

[23] Rao R, Bhattacharya N, Thomas N, Duan Y, Chen X, Canny J, Abbeel P, Song YS. Evaluating protein transfer learning with TAPE. Adv Neural Inf Process Syst. 2019;32:9689–9701. PMC7774645 33390682

[24] Xiao Y, Qiu J, Li Z, Hsieh C-Y, Tang J. Modeling protein using large-scale pretrain language model, 2021. arXiv preprint arXiv:2108.07435.

[25] Hie BL, Yang KK, Kim PS. Evolutionary velocity with protein language models; 2021. bioRxiv. 10.1016/j.cels.2022.01.003 35120643

[26] Zaremba W, Sutskever I, Vinyals O. Recurrent neural network regularization, 2014. arXiv preprint arXiv:1409.2329.

[27] Oord Avd, Dieleman S, Zen H, Simonyan K, Vinyals O, Graves A, Kalchbrenner N, Senior A, Kavukcuoglu K. Wavenet: A generative model for raw audio; 2016. arXiv preprint arXiv:1609.03499.

[28] Kim HY, Kim D (2020). Prediction of mutation effects using a deep temporal convolutional network. Bioinformatics.

[29] Goodfellow I. Nips 2016 tutorial: Generative adversarial networks; 2016. arXiv preprint arXiv:1701.00160.

[30] Creswell A, White T, Dumoulin V, Arulkumaran K, Sengupta B, Bharath AA (2018). Generative adversarial networks: an overview. IEEE Signal Process Mag.

[31] Radford A, Metz L, Chintala S. Unsupervised representation learning with deep convolutional generative adversarial networks; 2015. arXiv preprint arXiv:1511.06434.

[32] Zhu J-Y, Park T, Isola P, Efros AA. Unpaired image-to-image translation using cycle-consistent adversarial networks; 2017. In: proceedings of the IEEE international conference on computer vision, pp. 2223–2232.

[33] Karras T, Laine S, Aila T. A style-based generator architecture for generative adversarial networks. In: proceedings of the IEEE/CVF conference on computer vision and pattern recognition, pp. 2019:4401–4410.

[34] Repecka D, Jauniskis V, Karpus L, Rembeza E, Rokaitis I, Zrimec J, Poviloniene S, Laurynenas A, Viknander S, Abuajwa W (2021). Expanding functional protein sequence spaces using generative adversarial networks. Nat Mach Intell.

[35] Sinai S, Kelsic E, Church GM, Nowak MA. Variational auto-encoding of protein sequences; 2017. arXiv preprint arXiv:1712.03346.

[36] Greener JG, Moffat L, Jones DT (2018). Design of metalloproteins and novel protein folds using variational autoencoders. Sci Rep.

[37] Hawkins-Hooker A, Depardieu F, Baur S, Couairon G, Chen A, Bikard D (2021). Generating functional protein variants with variational autoencoders. PLoS Comput Biol.

[38] Kingma DP, Welling M. Auto-encoding variational bayes; 2013. arXiv preprint arXiv:1312.6114.

[39] Mistry J, Chuguransky S, Williams L, Qureshi M, Salazar GA, Sonnhammer EL, Tosatto SC, Paladin L, Raj S, Richardson LJ (2021). Pfam: the protein families database in 2021. Nucleic Acids Res.

[40] Shang Y, Huang S (2020). Engineering plant cytochrome p450s for enhanced synthesis of natural products: past achievements and future perspectives. Plant Commun.

[41] Romero PA, Krause A, Arnold FH (2013). Navigating the protein fitness landscape with gaussian processes. Proc Natl Acad Sci.

[42] Li Y, Drummond DA, Sawayama AM, Snow CD, Bloom JD, Arnold FH (2007). A diverse family of thermostable cytochrome p450s created by recombination of stabilizing fragments. Nat Biotechnol.

[43] Ezkurdia I, Tress ML (2011). Protein structural domains: definition and prediction. Curr Protoc Protein Sci.

[44] Veretnik S, Shindyalov I. Computational methods for domain partitioning of protein structures. In: computational methods for protein structure prediction and modeling, 2007:125–145.

[45] Fujiwara Y, Asogawa M (2001). Prediction of subcellular localizations using amino acid composition and order. Genome Inform.

[46] Almagro Armenteros JJ, Sønderby CK, Sønderby SK, Nielsen H, Winther O (2017). Deeploc: prediction of protein subcellular localization using deep learning. Bioinformatics.

